# Impact of the COVID‐19 pandemic on acute stroke care: An analysis of the 24‐month data from a comprehensive stroke center in Shanghai, China

**DOI:** 10.1111/cns.14148

**Published:** 2023-03-08

**Authors:** Qimin Hu, Yiming Hu, Yue Gu, Xiaoyan Song, Yijue Shen, Haiyan Lu, Li Zhang, Peifeng Liu, Guodong Wang, Chunni Guo, Kan Fang, Qiaoshu Wang

**Affiliations:** ^1^ Department of Neurology Shanghai General Hospital, Shanghai Jiao Tong University School of Medicine Shanghai China

**Keywords:** COVID‐19, emergency service, hospital, ischemic stroke, pandemics

## Abstract

**Introduction:**

Whether the coronavirus disease‐2019 (COVID‐19) pandemic is associated with a long‐term negative impact on acute stroke care remains uncertain. This study aims to compare the timing of key aspects of stroke codes between patients before and after the COVID‐19 pandemic.

**Methods:**

This retrospective cohort study was conducted at an academic hospital in Shanghai, China and included all adult patients with acute ischemic stroke hospitalized via the emergency department (ED) stroke pathway during the 24 months since the COVID‐19 outbreak (COVID‐19: January 1, 2020–December 31, 2021). The comparison cohort included patients with ED stroke pathway visits and hospitalizations during the same period (pre‐COVID‐19: January 1, 2018–December 31, 2019). We compared critical time points of prehospital and intrahospital acute stroke care between patients during the COVID‐19 era and patients during the pre‐COVID‐19 era using *t* test, χ^2^, and Mann–Whitney *U* test where appropriate.

**Results:**

A total of 1194 acute ischemic stroke cases were enrolled, including 606 patients in COVID‐19 and 588 patients in pre‐COVID‐19. During the COVID‐19 pandemic, the median onset‐to‐hospital time was about 108 min longer compared with the same period of pre‐COVID‐19 (300 vs 192 min, *p* = 0.01). Accordingly, the median onset‐to‐needle time was 169 min in COVID‐19 and 113 min in pre‐COVID‐19 (*p* = 0.0001), and the proportion of patients with onset‐to‐hospital time within 4.5 h was lower (292/606 [48.2%] vs 328/558 [58.8%], *p* = 0.0003) during the pandemic period. Furthermore, the median door‐to‐inpatient admission and door‐to‐inpatient rehabilitation times increased from 28 to 37 h and from 3 to 4 days (*p* = 0.014 and 0.0001).

**Conclusions:**

During the 24 months of COVID‐19, a prolongation of stroke onset to hospital arrival and to intravenous rt‐PA administration times were noted. Meanwhile, acute stroke patients needed to stay in the ED for a longer time before hospitalization. Educational system support and process optimization should be pursued in order to acquire timely delivery of stroke care during the pandemic.

## Graphical abstract

During the 24 months of COVID‐19 (January 1, 2020–December 31, 2021), a prolongation of stroke onset to hospital arrival and to intravenous rt‐PA administration times were noted. These findings suggest that COVID‐19 has imposed a largely negative impact on acute stroke care in China since the COVID‐19 pandemic.

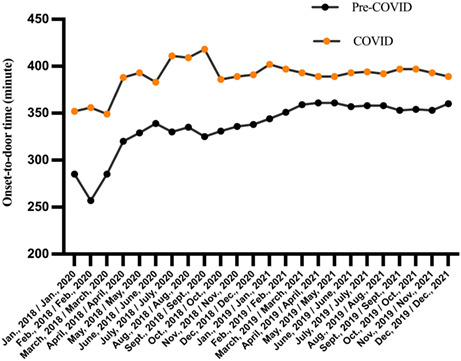

## 1 INTRODUCTION

Ischemic stroke is a devastating cerebrovascular disease with serious adult disability and mortality worldwide. Acute ischemic stroke (AIS) management relies on timely delivery. The evaluation of a patient and a decision to treatment, that is, within different time windows after acute stroke onset, need to be made effectively.^1^, ^2^, ^3^, ^4^ The coronavirus disease 2019 (COVID‐19) outbreak has affected several aspects of acute stroke care since its emergence in December 2019 in China, including declines in the number of stroke patients, delays in stroke onset to hospital arrival time.^5^, ^6^, ^7^, ^8^, ^9^

Since the first case of COVID‐19 was confirmed in Shanghai on January 20, 2020, strict measures were taken to prevent and control the spread of COVID‐19.^10^ During the period of lockdown in Shanghai (January 24 to March 24, 2020), the number of outpatient and emergency department visits was dropped dramatically compared with the same period of 2019.^11^, ^12^ Different from western countries, China has implemented the normalized epidemic prevention and control and zero‐COVID policy since the COVID‐19 outbreak. In terms of hospital admission during the pandemic, official guidelines were launched in Shanghai that it is mandatory to perform epidemiological investigation, COVID‐19 nucleic acid detection, and Chest CT before in‐hospital management.^10^ Several studies showed that during the early lockdown phase of the pandemic, the COVID‐19 outbreak impacted stroke care significantly in China, including a prolongation in stroke onset to hospital arrival time, a significant drop in admissions, thrombolysis, and thrombectomy.^7^, ^9^, ^12^ However, it remains not clear whether the pandemic has a long‐term effect on the timing of key aspects of the in‐hospital stroke pathway. In the present study, we, therefore, compared the onset to hospital arrival (onset‐to‐door) time, onset to administration of intravenous recombinant tissue plasminogen activator [rt‐PA] (onset‐to‐needle) time, door‐to‐needle time, door‐to‐inpatient admission time, and other critical time points of AIS during the COVID‐19 pandemic from January 2020 to December 2021 with a corresponding period in 2018 and 2019.

## 2 MATERIALS AND METHODS

### 2.1 Design

This single‐center retrospective cohort study was conducted at the Southern Campus of the Shanghai General Hospital, which is one of the 46 large hospitals in Shanghai with over 2000 beds and is also the sole academic tertiary hospital with a comprehensive stroke center to the population of 2,000,000.^13^ One group of the study population comprised consecutive patients 18 years or older who had confirmation of acute ischemic stroke (AIS) by a panel of attending neurologists and had an acute stroke pathway visit in emergency department (ED) and hospitalization from January 1, 2020 to December 31, 2021. In parallel, we enrolled adult patients with an ED visit and hospitalization with AIS during the same period in 2018 and 2019 (pre‐COVID‐19: January 1, 2018–December 31, 2019). Available data were retrieved from the Shanghai General Hospital stroke registry, which is approved by the local institutional review board and waived the requirement for informed consent.

### 2.2 Measurements

The acute stroke pathway comprises a stroke multidisciplinary team that enrolls patients presenting to the ED within 24 h of stroke onset, including all acute stroke patients transferred by ambulance, coming to the hospital by themselves, or transferred from outpatient service. The diagnosis of AIS was confirmed by a panel of attending neurologists. Exclusion criteria were the following: any stroke code that after initial evaluation from the ED stroke pathway was determined not to be a stroke, any stroke patients admitted to Shanghai General Hospital without going through the acute stroke pathway, any stroke transferred to the hospital when the stroke presenting >24 h after symptoms onset. Stroke onset was defined as the last time the patient was observed without deficit or last seen well time.

We used electronic data capture and manual abstraction to collect information from our clinical research database, including demographics, vascular risk factors, onset‐to‐door time (ODT), onset‐to‐needle time (ONT), door‐to‐needle time (DNT), door‐to‐inpatient admission time (DAT), door‐to‐inpatient rehabilitation time (DRT), and details of AIS treatment.

Stroke onset‐to‐door time was defined as the duration between stroke onset to ED arrival; onset‐to‐needle time was the duration between stroke onset to administration of IV rt‐PA (intravenous recombinant tissue‐type plasminogen activator); door‐to‐needle time was the duration between ED arrival to the administration of IV rt‐PA; door‐to‐inpatient admission time was the duration between ED arrival to admitted to the inpatient setting, whereas door‐to‐inpatient rehabilitation time was the time between ED arrival to initiation of rehabilitation.

### 2.3 Analysis

We analyzed baseline demographics, vascular risk factors, and critical time points between AIS patients during COVID‐19 and those pre‐COVID‐19 using *t* test or Mann–Whitney *U* test (not normally distributed) for continuous variables and *χ* ^*2*^ for categorical variables with two‐sided *p* values to show significance, for cell <5, Fisher exact test was used. Normality of continuous variables was tested using the Shapiro–Wilk test. Statistical analyses were performed with Prism 7.0d (GraphPad Software Inc). A two‐sided *α* level of 0.05 was used to show a statistically significant difference.

## 3 RESULTS

### 3.1 Participants

**FIGURE 1:**
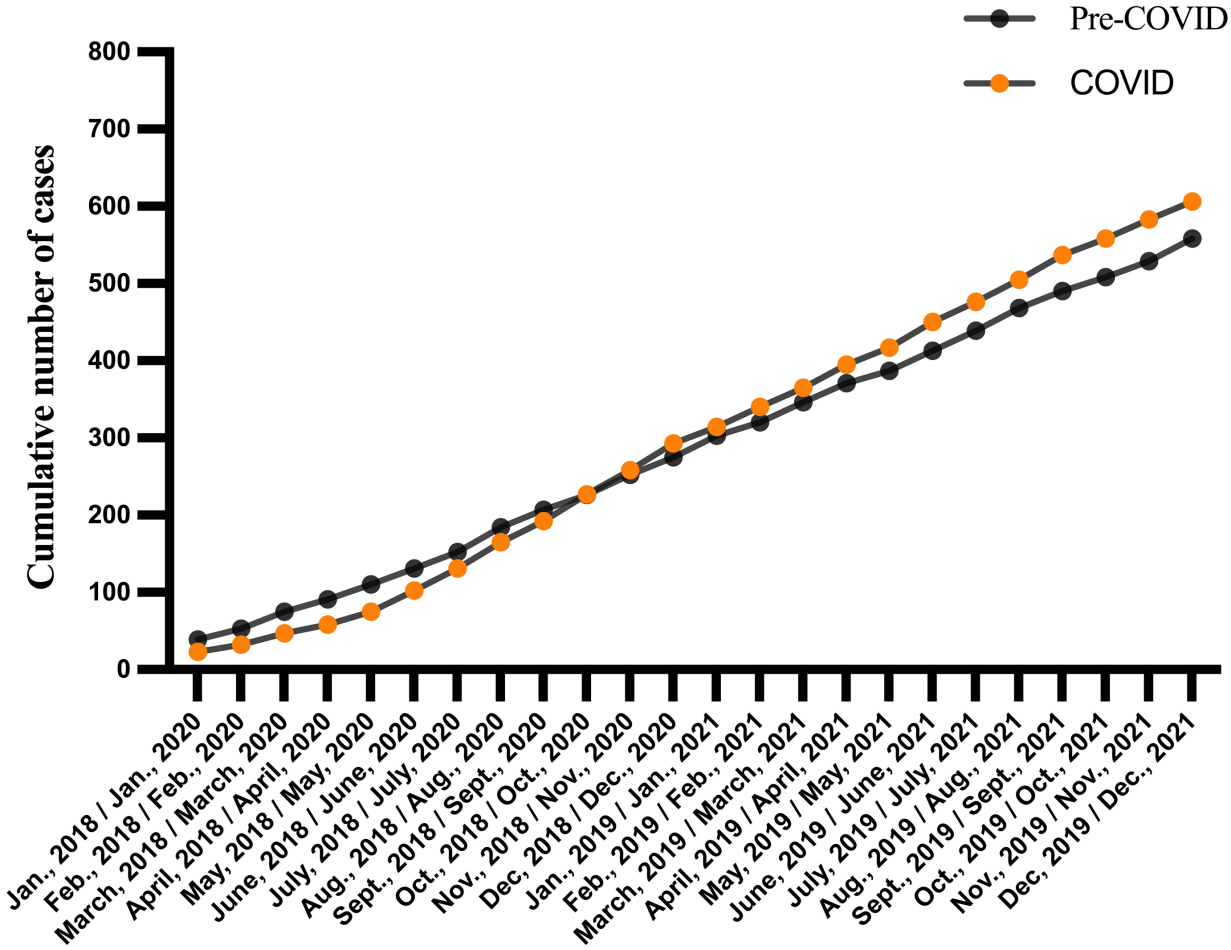
Cumulative number of acute ischemic stroke cases during the coronavirus disease 2019 (COVID‐19) and the pre‐COVID periods. The COVID‐19 period: January 1, 2020–December 31, 2021; the pre‐COVID period: January 1, 2018–December 31, 2019.

**FIGURE 2:**
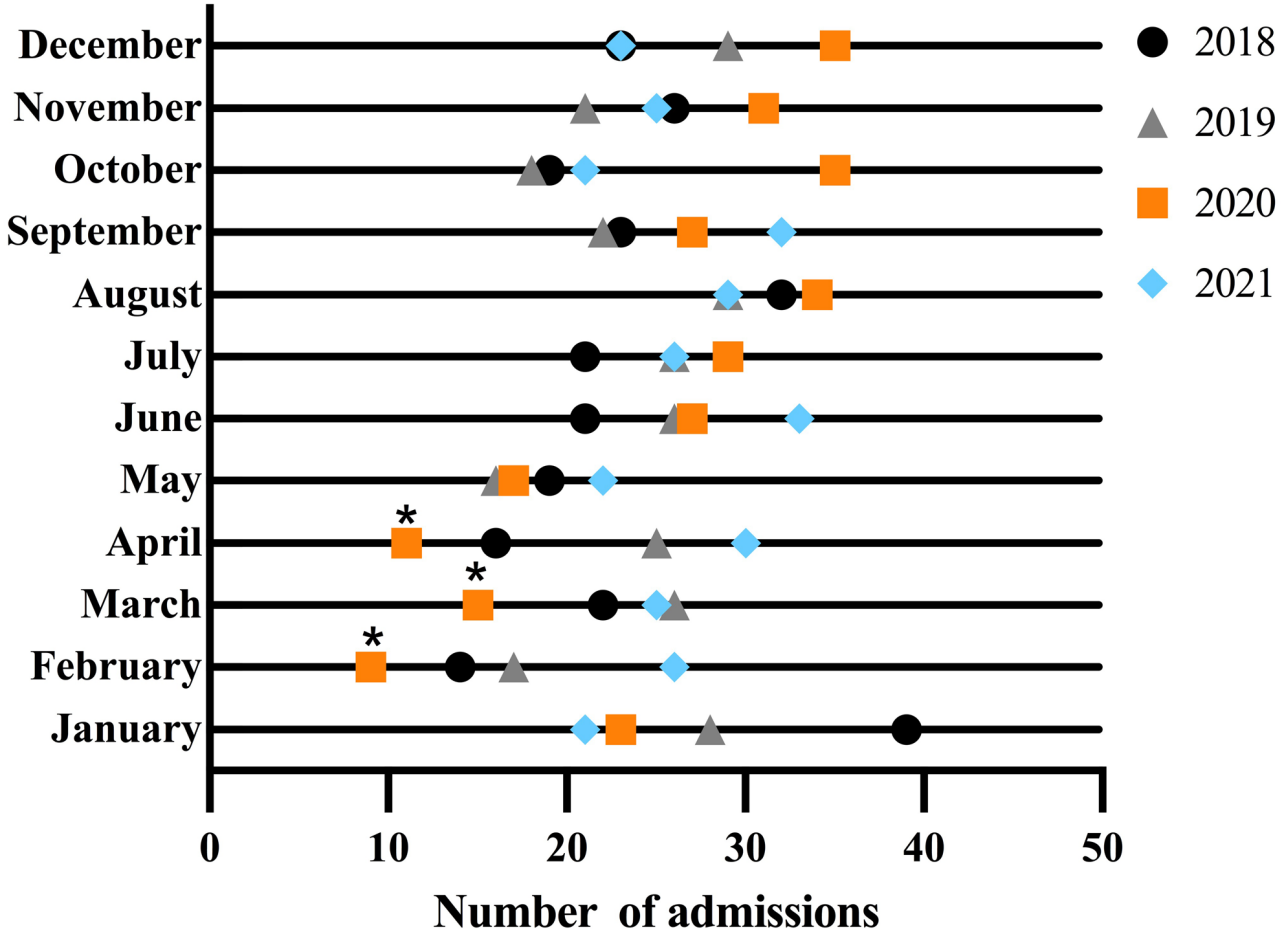
Monthly number of acute ischemic stroke cases during the coronavirus disease‐2019 (COVID‐19) and the pre‐COVID periods. The COVID‐19 period: January 1, 2020–December 31, 2021; the pre‐COVID period: January 1, 2018–December 31, 2019. *Nadirs of 1164 acute ischemic stroke hospitalizations in the 3‐month period at the height of the pandemic (February 1 to April 31, 2020).

During the period January 1, 2018–December 31, 2021, a total of 1194 acute ischemic stroke cases that met the criteria were hospitalized via ED stroke pathway. There were 606 patients in the COVID‐19 pandemic (January 1, 2020–December 31, 2021) and 588 patients in the pre‐COVID‐19 period (January 1, 2018–December 31, 2019). There was nearly an 8.6% increase in the total number of hospitalizations via acute stroke pathway in the COVID‐19 pandemic period, and the cumulative number of patients dropped from January to September 2020 and continuously increased from October 2020 to December 2021, compared with the same period in 2018 and 2019 (Figure 1). The annual number of admitted cases increased gradually from 2018 (275) and 2019 (283, 2.9% increase) to 2020 (293, 3.5% increase) and 2021 (313, 6.8% increase) (Table 1). The COVID‐19 lockdown in Shanghai was from January 24 to March 24, 2020, therefore, we analyzed the impact on stroke patient admissions in a 3‐month period at the height of the pandemic (February 1–April 31, 2020). The hospitalization volumes for acute ischemic stroke were 17, 26, and 25 in these 3 months of 2019 versus 9, 15, and 11 during the same period of 2020, representing declines of 47.1%, 42.3%, and 56.0%, respectively (Table 1). When the lockdown measures were progressively eased from March 24, 2020, the number of cases gradually returned and even exceeded the previous level (Figure 2).

**TABLE 1:** Changes in stroke admissions and thrombolysis from 2018 to 2021.

	No. of Admissions and Thrombolysis			
	2018	2019	2020	2021
Total No. of Admissions	275	283	293	313
Percentage of change		2.9%	3.5%	6.8%
February	14	17	9	26
Percentage of change		21.4%	−47.1%	188.9%
March	22	26	15	25
Percentage of change		18.1%	−42.3%	66.6%
April	16	25	11	30
Percentage of change		56.3%	−56.0%	172.7%
Total No. of thrombolysis	42	56	71	75
Percentage of change		33.3%	26.8%	5.6%
February	6	1	2	8
March	1	5	2	6
April	3	5	1	12
Total No. of thrombolysis from February to April	9	11	5	26
Percentage of change		22.2%	−54.5%	420%

During the COVID‐19 period, 38.9% of the patients (*n* = 236) had diabetes, while 31.7% of the patients (*n* = 177) had diabetes during the pre‐COVID‐19 period (*p* = 0.01). Patients with atrial fibrillation were 30 (5.0%) in the COVID‐19 group and 44 (8.2%) in the pre‐COVID‐19 group (*p* = 0.02). NIHSS scores were greater in the pre‐COVID‐19 group than that in the COVID‐19 group. No significant differences were noted in age, sex, and vascular risk factors between the two groups (Table 2).

**TABLE 2:** Characteristics of patients with acute ischemic stroke, stratified by the COVID‐19 pandemic.

	Pre–COVID‐19 (*n* = 558)	COVID‐19 (*n* = 606)	*p* value
Demographic			
Age, median (IQR), year	63.0 (55.0–70.0)	64.0 (55.0–71.0)	0.44
Male, %	404 (72.4)	439 (72.4)	0.99
Vascular risk factors			
Ever smokers, %	250 (44.8)	251 (41.4)	0.24
Hypertension, %	404 (72.4)	462 (76.2)	0.13
Diabetes, %	177 (31.7)	236 (38.9)	0.01
LDL (mmol/L), median (IQR)	2.53 (1.97–3.13)	2.46 (1.85–3.09)	0.21
Atrial fibrillation, %	46 (8.2)	30 (5.0)	0.02
Clinical characteristics			
NIHSS, median (IQR)	3 (1–6)	3 (1–4)	0.002
Onset‐to‐door time, median (IQR), min	192 (90–480)	300 (112–540)	0.01
Onset‐to‐door ≤6 h, %	382 (65.0)	380 (62.7)	0.43
Onset‐to‐door ≤4.5 h, %	328 (58.8)	292 (48.2)	0.0003
Intravenous thrombolysis，%	98 (17.6)	146 (24.1)	0.006
Onset‐to‐needle time, median (IQR),min	113 (80–150)	169 (135–204)	0.0001
Door‐to‐needle time, median (IQR),min	69 (53–89)	71 (57–88)	0.48
Door‐to‐needle time ≤ 60 min, %	30 (30.6)	44 (30.1)	0.99
Door‐to‐CT time before IV rt‐PA, median	17 (10–26)	19 (14–30)	0.012
(IQR), min			
CT‐to‐needle time, median (IQR),min	50 (38–66)	48 (40–59)	0.52
Door‐to‐inpatient admission time,	28 (18–68)	37 (21–71)	0.014
Median (IQR), hour			
Door‐to‐inpatient rehabilitation time, median (IQR), day	3 (2–5)	4 (2–6)	0.0001

Abbreviations: COVID‐19, coronavirus disease‐2019; IQR, interquartile range; IV rt‐PA, intravenous recombinant tissue‐type plasminogen activator; LDL, low‐density lipoprotein; NIHSS, National Institutes of Health Stroke Scale.

### 3.2 Comparison of the COVID‐19 and the pre‐COVID‐19 Cohorts

**FIGURE 3:**
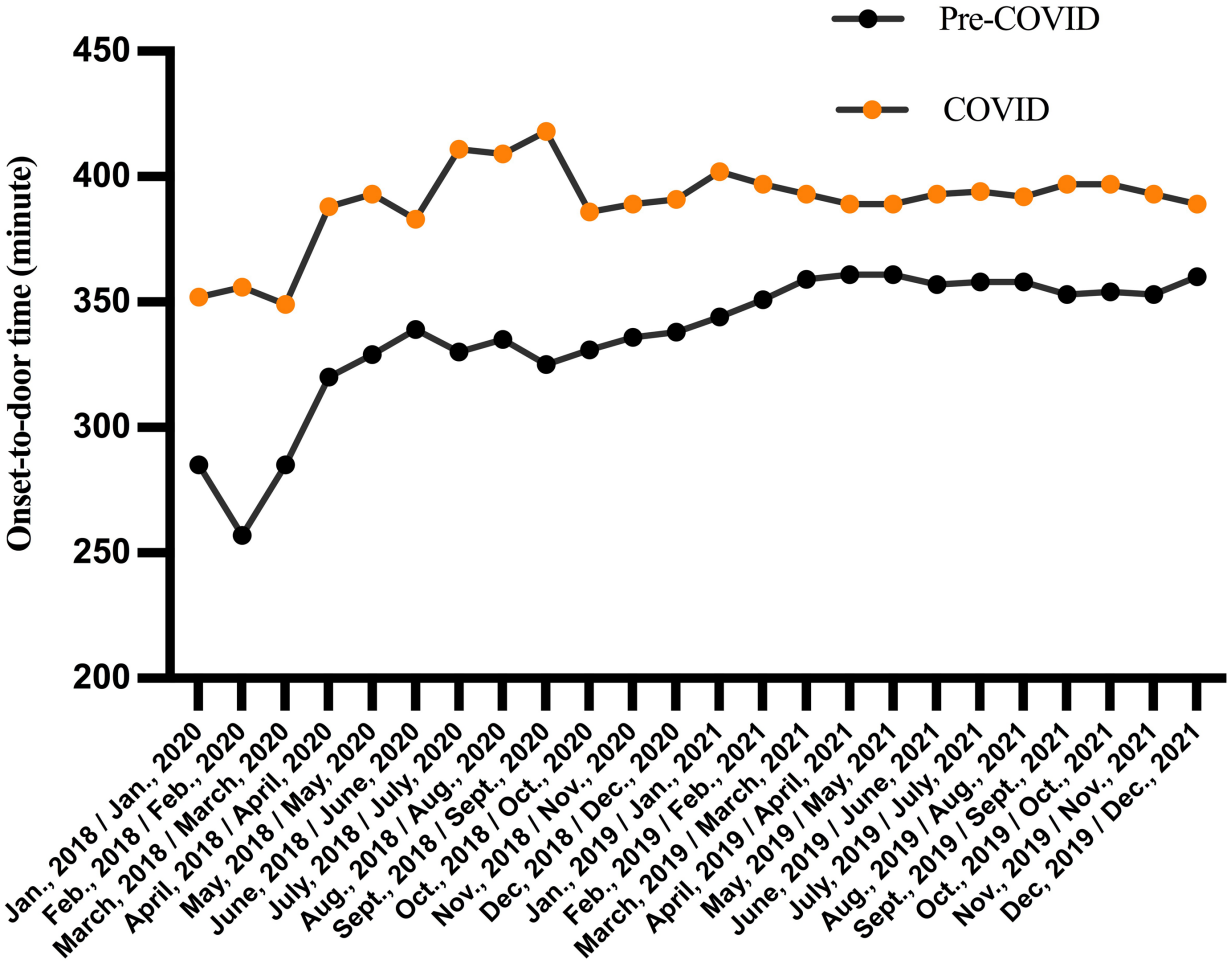
The mean of onset‐to‐door time during the coronavirus disease‐2019 (COVID‐19) and the pre‐COVID periods. The COVID‐19 period: January 1, 2020–December 31, 2021; the pre‐COVID period: January 1, 2018–December 31, 2019.

Compared with the pre‐COVID‐19 group, the onset‐to‐door time was longer (300 [112–540] min vs 192 (90–480) min, *p* = 0.01) in the COVID group. We further analyzed the changes in ODTs during different periods of the pandemic. The results showed that the ODTs were significantly extended from April 2020 and peaked in September 2020 and then maintained at a level higher than that before the pandemic (Figure 3). During the COVID‐19 period, 62.7% (380/606) of patients with acute cerebral infarction arrived at the hospital within 6 h, which is similar to the proportion of 65.0% (382/558) patients before the pandemic (*p* = 0.43). Otherwise, the proportion of patients who arrived within 4.5 h during the COVID‐19 period was significantly less than that during the pre‐COVID‐19 period (292/606 [48.2%] vs 328/558 [58.8%], *p* = 0.0003), and the proportion of patients with intravenous thrombolysis was significantly higher (146/606 [24.1%] vs 98/558 [17.6%], *p* = 0.006) during the pandemic. Onset‐to‐needle time in the COVID‐19 group was significantly longer than that in the pre‐COVID‐19 group (169 [135–204] min vs 113 [80–150] min, *p* = 0.0001).

Our findings showed that during the COVID‐19 period, there was no significant delay in the acute stroke pathway specifically in the time from door to rt‐PA administration. The median stroke door‐to‐needle time during the COVID‐19 period was similar to that during the pre‐COVID‐19 period (71 [57–88] min vs 69 [53–89] min, *p* = 0.48). The proportion of DNT less than 60 min was 30.6% (30/98) before COVID‐19 and 30.1% (44/146) during COVID‐19 (*p* = 0.99). But we found the door‐to‐CT time before IV rt‐PA in COVID‐19 was longer than that in pre‐COVID‐19 (19 [14–30] min vs 17 [10–26] min, *p* = 0.012). Furthermore, we identified significant delays the door‐to‐inpatient admission time and the door‐to‐inpatient rehabilitation time. The median DAT was 37 h (IQR, 21–71) in patients during the COVID‐19 pandemic vs 28 h (IQR, 18–68) in patients during the pre‐COVID‐19 period (*p* = 0.014). The median DRT was considerably longer during the period of COVID‐19 compared with that during the pre‐COVID‐19 period (*p* = 0.0001, Table 2).

## 4 DISCUSSION

In this retrospective cohort study, our findings suggest the possible delays of stroke care in patients with ED visits and hospitalizations during the COVID‐19 period. Compared with the same period in 2018 and 2019, the median symptom onset‐to‐door time was up to 108 min long and the proportion of patients arriving within the 4.5 h time window of IV rt‐PA was significantly lower during the COVID‐19 pandemic. The median stroke onset‐to‐needle time was 56 min longer, and the median door‐to‐admission time and door‐to‐rehabilitation time was 9 h and 1 day longer during the COVID‐19 pandemic.

Growing evidence indicates a nonnegligible prevalence of neurological involvement during COVID‐19 infection or postinfection period, including seizures, encephalopathy, acute ischemic stroke, etc.^14^, ^15^, ^16^, ^17^ The systematic review of 100,949 patients with COVID‐19 from the early phase of the pandemic to the omicron waves reported a pooled incidence of acute ischemic stroke in patients hospitalized for COVID‐19 of 0.9%.^18^ Though the frequency seems low, concomitant COVID‐19 infection and stroke might result in poor outcomes despite aggressive medical treatment, suggesting an urgency for timely diagnosis and emergency treatment of COVID‐19‐associated stroke.^16^ Paradoxically, there was a global decline in new stroke admissions and reperfusion therapies at the height of the pandemic.^9^, ^19^ Analysis of time metrics in the stroke pathway may help identify the underlying reasons why stroke rates declined in contrast to expected neurological complications related to COVID‐19 and enhance procedures on acute ischemic stroke management in preparation for the potential increase of new‐onset stroke patients.

Unlike other countries with a heavy burden of COVID‐19 hospitalization, the number of confirmed COVID‐19 cases per month in Shanghai rarely exceeded 30 during the pandemic period of 2020 and 2021.^10^, ^19^ In Shanghai, the drop in the absolute number of hospitalizations and thrombolysis in the early stage of the COVID‐19 outbreak in 2020 is most likely because of the lockdown with strong containment measures. Based on Table 1 for the data from pre‐COVID‐19 (2018 and 2019) to COVID‐19 (2000 and 2021), there were 58 (606–558) more AIS hospitalizations. Hence, our study reflects the impact of long‐term stringent COVID‐19 epidemic prevention and control measures and zero‐COVID policy on acute stroke care. We have demonstrated that the onset‐to‐door and onset‐to‐needle times were significantly prolonged during the COVID‐19 pandemic.

For acute ischemic stroke, it is widely accepted that the wise strategy is to obtain earlier reperfusion, which is associated with better clinical outcomes.^20^ A decision to treatment should be made timely, including tissue plasminogen activator (t‐PA) that can only be given in the first 4.5 h after symptom onset and mechanical thrombectomy to be performed within 24 h of acute ischemic stroke.^4^, ^20^, ^21^, ^22^ Any delays in seeking those treatments may exert a negative impact on stroke outcomes, including the short‐term functional recovery and the long‐term outcomes of death and recurrent ischemic stroke.^20^, ^23^ A multicenter, hospital‐based study that included 6780 consecutive patients with ischemic stroke demonstrated that early hospital arrival within 6 h of stroke onset was associated with early neurological improvement and favorable functional outcome after 90 days, regardless of reperfusion treatment or the severity of the stroke.^24^

Our results align with a recent report in Hong Kong, China, showing a prolongation of stroke onset‐to‐door time, identifying a significant reduction of hospital arrival within 4.5 h during the first 60 days since the first diagnosed COVID‐19 case (COVID‐19: January 23, 2020–March 24, 2020).^7^ When the main inclusion criterion was narrowed to stroke patients who received reperfusion treatments, the prehospital time in the 2020 group was still significantly longer than that in the 2019 group, as revealed by an investigation among 8 certified stroke hospitals in Huizhou, China from January 1, 2020 to May 31, 2020.^25^ Besides, there was a population‐based cross‐sectional study about the association of prehospital delay and the stroke educational campaign conducted before the pandemic in an adjacent district in Shanghai, China, enrolling patients presented within 2 days of stroke onset.^26^ By comparison, the onset to door time in our pre‐COVID‐19 group is roughly equivalent to that in the postcampaign period, whereas the time from onset to door during the pandemic tends to return to the level before the stroke educational campaign.^26^ Since the pandemic has been going on for a long period of time, focusing on the early stages is insufficient for a comprehensive presentation. There is still a necessary demand for research with a longer study period and larger sample sizes to reflect the impact of COVID‐19 on stroke onset‐to‐door time as a whole.

Based on our data (Figure 3), the extension of onset‐to‐door time has lasted 2 years since the outbreak of the pandemic. We believe that there are several reasons for the prolongation of the time from onset to arrival at the hospital. On the one hand, during the pandemic, in the acute phase of stroke patients may be reluctant to come to the hospital due to fear of the virus.^9^ Under the “dynamic zero‐COVID policy,” people in China had a higher level of anxiety, compared with that of people living in Western countries.^27^ In a retrospective observational cohort study performed in Shanghai, there was nearly a 30% drop in the total number of primary care general practice consultation visits in the early stage of the COVID‐19 pandemic from January to June 2020, compared to the same period in 2019.^11^ On the other hand, in Shanghai, during the COVID‐19 period, some primary stroke centers were closed or unable to treat stroke patients for several months due to the need of pandemic prevention.^28^ In an observational study in China, a survey distributed to the leaders of stroke centers in 280 hospitals, indicated that during the COVID‐19 outbreak in 2020, 70% of hospitals reduced their emergency stroke capacity, and over 4% of stroke centers were closed.^12^ As a regional medical center with no change in stroke capacity, our hospital admitted more stroke patients (8.6% increase) during the pandemic. Additionally, at the early stage of the COVID‐19 outbreak, patient transportation was limited due to the lockdown. Thereafter, under the “dynamic zero‐COVID policy,” some hospitals with stroke centers were closed for several days or a few months without warning due to COVID‐19‐positive patients detected in these institutions. Thus, the passive adjustment of medical transfer system may lead to the reduction of transportation efficiency and the prolongation of onset to hospital arrival.

Previous studies indicated that patients who were treated with tPA and had door‐to‐needle times of longer than 60 min, had significantly higher all‐cause mortality, higher symptomatic intracranial hemorrhage, and lower odds of independent ambulation, compared with those treated within 60 min.^29^, ^30^ Since the COVID‐19 pandemic outbreak, “dynamic zero‐COVID policy” has been implemented in China, which requires continuous adjustment and improvement of the medical system. For identification of COVID‐19‐positive patients, in ED acute stroke pathway all patients are required to undergo the COVID‐19 screening process, including the epidemiological investigation and chest computed tomography (CT). These measures were time‐consuming and occupied CT scanners, leading to delayed stroke imaging and therapy. Our study indicated that the median door‐to‐CT time was longer about 2 min during the COVID‐19 pandemic in comparison with the pre‐COVID‐19 period, which may be attributed to the extra time needed to do the epidemiological investigation and chest CT. Otherwise, we found that the door‐to‐needle times were not prolongated significantly in the COVID‐19 group and the proportion of door‐to‐needle time within 60 minute was similar between the two periods, which may be owed to decreased CT‐to‐needle time and the process improvement during the pandemic.

In terms of the door‐to‐inpatient admission time and door‐to‐inpatient rehabilitation time, our data suggest that there were 9 h and 1 day longer median time in AIS patients admitted to hospitals during the COVID‐19 pandemic. Several studies demonstrated that earlier transfer to an inpatient rehabilitation setting was associated with better functional improvement after stroke.^31^, ^32^ Our data showed that the COVID‐19 pandemic may have a negative impact on stroke recovery. There are several factors that may interfere with the routine admission process of AIS patients. During the pandemic, medical resources were reorganized in primary medical centers, and more AIS patients were transferred to academic hospitals compared with pre‐COVID‐19 period, which led to prolonged door‐to‐admission time. Based on our data (Table 1), compared with that before the pandemic, the annual growth rate of patients admitted to hospitals has continued to increase, which can partly explain the extension of waiting time for admission. Additionally, several COVID‐19‐related investigations were scrutinized before admission according to the official guidelines, especially nucleic acid testing of COVID‐19 within 48 h or 24 h which is needed for all patients and their families who want to enter the inpatient setting. Furthermore, during the pandemic, it is mandated for the medical affair department to review the results of COVID‐19 test and chest CTs to determine whether patients in the ED stroke pathway are suitable for in‐hospital treatment. All those measures are time‐consuming, and keep AIS patients in the ED stroke pathway for a longer time.

There are several strengths of this study. Our study is the largest cohort of patients arriving at the hospital within 24 h after stroke onset during the long‐term COVID‐19 pandemic in China. It also includes multidimensional in‐hospital time points, providing a more nuanced view of factors that affect stroke service. Moreover, in China, due to the strong control of the central government over local governments, the epidemic prevention policies of various regions are uniform and thus given the geographic distribution and inclusion of an academic hospital that covers a population of 2,000,000, the findings are representative of the impact of the COVID‐19 on stroke care in China for patients with acute cerebral infarction.

### 4.1 Limitations

Our study has several limitations. First of all, the main limitation of our study is its retrospective nature with potential selection bias in nonrandomized data. Second, due to different stroke rescue processes in different hospitals, the results of our single‐center study may not be generalizable to other settings. Patients treated with mechanical thrombectomy were not included in our analysis due to the small number of cases before the pandemic period. In particular, during the lockdown, under strict containment some patients with undiagnosed strokes may have died before reaching the hospital, therefore, we were unable to evaluate the population‐level impact of COVID‐19 among patients with acute ischemic stroke. Third, during the COVID‐19 period, stroke epidemiology may be changed, and part of patients' behavior may be altered—that they need more time to evaluate the risk and benefits of being in‐hospital, fearing of being exposed to the virus and inconvenience caused by quarantine measures.^33^ In addition, with the change in pandemic situation and the emergence of new COVID variants, the prevention and control policies are also changing, which will also affect the patient's medical behavior.

## 5 CONCLUSIONS

The onset‐to‐door time, onset‐to‐needle time, and door‐to‐inpatient admission time of patients with AIS in ED stroke pathway visits during the long‐term COVID‐19 pandemic (2020 and 2021) was longer than those of patients who visited the ED during the pre‐COVID‐19 period (2018 and 2019). These findings suggest that COVID‐19 has imposed a largely negative impact on acute stroke care in China since the COVID‐19 pandemic, thus updated stroke guidelines suitable to tackle the double stress of the pandemic and policy are necessary to prompt the improvement of emergency management of acute stroke.

## AUTHOR CONTRIBUTIONS

Drs Qiaoshu Wang and Kan Fang have full access to all the data in the study and take responsibility for the integrity of the data and the accuracy of the data analysis. Drs Qimin Hu, Yiming Hu, and Yue Gu are co‐first authors. *Concept and design:* Q. Wang, K. Fang, and Q. Hu. *Acquisition, analysis, or interpretation of data:* Q Hu, Y Hu, Gu, Song, Shen, Lu, L. Zhang, Liu, G Wang, Guo, Fang, and Q Wang. *Drafting of the manuscript:* Q Wang, Fang, Y Hu, and Gu. *Critical revision of the manuscript for important intellectual content:* Q Hu, Y Hu, Gu, Song, Shen, Lu, L Zhang, Liu, G Wang, Guo, Fang, and Q Wang. *Statistical analysis:* Q Wang, Gu, and Y Hu. *Administrative, technical, or material support:* Q Hu, Y Hu, Gu, Song, Fang, and Q Wang. *Supervision:* Q Wang, Fang, and Q Hu.

## FUNDING INFORMATION

This study is funded by the Clinical Research Plan of Shanghai Hospital Development Center (16CR2046B), the National Natural Science Foundation of China (81371304), and the Shanghai Pujiang Program (15PJD031).

## CONFLICT OF INTEREST STATEMENT

The authors declare that they have no conflict of interest.

## PATIENT CONSENT STATEMENT

Informed consent was not sought for the present study because the manuscript does not contain patient data.

## PERMISSION TO REPRODUCE MATERIAL FROM OTHER SOURCES

Not Applicable.

## CLINICAL TRIAL REGISTRATION

Not Applicable.

## ROLE OF THE FUNDER/SPONSOR

The funding organizations had no role in the design and conduct of the study; collection, management, analysis, and interpretation of the data; preparation, review, or approval of the manuscript; and decision to submit the manuscript for publication.

## Data Availability

The data that support the findings of this study are available from the corresponding author upon reasonable request.

## References

[1] Caplan LR , Gorelick PB , Hier DB . Race, sex and occlusive cerebrovascular disease: a review. Stroke. 1986;17(4):648‐655. doi:10.1161/01.str.17.4.648 3526645

[2] Lees KR , Emberson J , Blackwell L , et al. Effects of alteplase for acute stroke on the distribution of functional outcomes: a pooled analysis of 9 trials. Stroke. 2016;47(9):2373‐2379. doi:10.1161/STROKEAHA.116.013644 27507856 PMC5024752

[3] Goyal M , Menon BK , van Zwam WH , et al. Endovascular thrombectomy after large‐vessel ischaemic stroke: a meta‐analysis of individual patient data from five randomised trials. Lancet. 2016;387(10029):1723‐1731. doi:10.1016/S0140-6736(16)00163-X 26898852

[4] Nogueira RG , Jadhav AP , Haussen DC , et al. Thrombectomy 6 to 24 hours after stroke with a mismatch between deficit and infarct. N Engl J Med. 2018;378(1):11‐21. doi:10.1056/NEJMoa1706442 29129157

[5] Dong E , Du H , Gardner L . An interactive web‐based dashboard to track COVID‐19 in real time. Lancet Infect Dis. 2020;20(5):533‐534. doi:10.1016/S1473-3099(20)30120-1 32087114 PMC7159018

[6] Lu H , Stratton CW , Tang YW . Outbreak of pneumonia of unknown etiology in Wuhan, China: the mystery and the miracle. J Med Virol. 2020;92(4):401‐402. doi:10.1002/jmv.25678 31950516 PMC7166628

[7] Teo KC , Leung WCY , Wong YK , et al. Delays in stroke onset to hospital arrival time during COVID‐19. Stroke. 2020;51(7):2228‐2231. doi:10.1161/STROKEAHA.120.030105 32432998 PMC7258759

[8] Kansagra AP , Goyal MS , Hamilton S , Albers GW . Collateral effect of Covid‐19 on stroke evaluation in the United States. N Engl J Med. 2020;383(4):400‐401. doi:10.1056/NEJMc2014816 32383831 PMC7233187

[9] Liu R , Zhao J , Fisher M . The global impact of COVID‐19 on acute stroke care. CNS Neurosci Ther. 2020;26(10):1103‐1105. doi:10.1111/cns.13442 32725844 PMC7539838

[10] Zhang N , Shi T , Zhong H , Guo Y . COVID‐19 prevention and control public health strategies in Shanghai, China. J Public Health Manag Pract. 2020;26(4):334‐344. doi:10.1097/PHH.0000000000001202 32433388

[11] Xu Z , Fan J , Ding J , et al. The impact of COVID‐19 on primary care general practice consultations in a teaching Hospital in Shanghai, China. Front Med (Lausanne). 2021;8:642496. doi:10.3389/fmed.2021.642496 33842504 PMC8033033

[12] Zhao J , Li H , Kung D , Fisher M , Shen Y , Liu R . Impact of the COVID‐19 epidemic on stroke care and potential solutions. Stroke. 2020;51(7):1996‐2001. doi:10.1161/STROKEAHA.120.030225 32432997 PMC7258753

[13] Chen Y , Wang L , Cui X , et al. COVID‐19 as an opportunity to reveal the impact of large hospital expansion on the healthcare delivery system: evidence from Shanghai, China. Ann Transl Med. 2021;9(16):1297. doi:10.21037/atm-21-2793 34532434 PMC8422135

[14] Vogrig A , Gigli GL , Bna C , Morassi M . Stroke in patients with COVID‐19: clinical and neuroimaging characteristics. Neurosci Lett. 2021;743:135564. doi:10.1016/j.neulet.2020.135564 33352277 PMC7749733

[15] Taquet M , Sillett R , Zhu L , et al. Neurological and psychiatric risk trajectories after SARS‐CoV‐2 infection: an analysis of 2‐year retrospective cohort studies including 1 284 437 patients. Lancet Psychiatry. 2022;9(10):815‐827. doi:10.1016/S2215-0366(22)00260-7 35987197 PMC9385200

[16] Dimitriadis K , Meis J , Neugebauer H , et al. Neurologic manifestations of COVID‐19 in critically ill patients: results of the prospective multicenter registry PANDEMIC. Crit Care. 2022;26(1):217. doi:10.1186/s13054-022-04080-3 35842675 PMC9287707

[17] Rothstein A , Favilla C , Sloane K , Witsch J . Perspective: COVID‐19 and its neurologic sequelae. Transl Perioper Pain Med. 2022;9(3):478‐481. doi:10.31480/2330-4871/162 PMC9645563 36381996

[18] Candeloro M , Schulman S . Arterial thrombotic events in hospitalized COVID‐19 patients: a short review and meta‐analysis. Semin Thromb Hemost. 2023;49(1):47‐54. doi:10.1055/s-0042-1749661 35793687

[19] Nogueira RG , Abdalkader M , Qureshi MM , et al. Global impact of COVID‐19 on stroke care. Int J Stroke. 2021;16(5):573‐584. doi:10.1177/1747493021991652 33459583 PMC8010375

[20] Prabhakaran S , Ruff I , Bernstein RA . Acute stroke intervention: a systematic review. Jama. 2015;313(14):1451‐1462. doi:10.1001/jama.2015.3058 25871671

[21] Hacke W , Kaste M , Bluhmki E , et al. Thrombolysis with alteplase 3 to 4.5 hours after acute ischemic stroke. N Engl J Med. 2008;359(13):1317‐1329. doi:10.1056/NEJMoa0804656 18815396

[22] Vinny PW , Vishnu VY , Padma Srivastava MV . Thrombectomy 6 to 24 hours after stroke. N Engl J Med. 2018;378(12):1161. doi:10.1056/NEJMc1801530 29565516

[23] Yafasova A , Fosbol EL , Johnsen SP , et al. Time to thrombolysis and long‐term outcomes in patients with acute ischemic stroke: a Nationwide study. Stroke. 2021;52(5):1724‐1732. doi:10.1161/STROKEAHA.120.032837 33657854

[24] Matsuo R , Yamaguchi Y , Matsushita T , et al. Association between onset‐to‐door time and clinical outcomes after ischemic stroke. Stroke. 2017;48(11):3049‐3056. doi:10.1161/STROKEAHA.117.018132 28974631

[25] Luo W , Li J , Li Z , Luo X , Chen M , Cai C . Effects of the COVID‐19 pandemic on reperfusion therapy for acute ischemic stroke patients in Huizhou City, China. Neurol Sci. 2021;42(2):467‐473. doi:10.1007/s10072-020-04938-w 33409830 PMC7787931

[26] Yuan J , Li M , Liu Y , et al. Analysis of time to the hospital and ambulance use following a stroke community education intervention in China. JAMA Netw Open. 2022;5(5):e2212674. doi:10.1001/jamanetworkopen.2022.12674 35579896 PMC9115614

[27] Shan D , Liu C , Li S , Zheng Y . Increased anxiety from fear of omicron in China as compared to North America and Western Europe: a cross‐sectional Kendall's tau‐b analysis using the generalized anxiety disorder 7‐item questionnaire. Front Psych. 2022;13:977361. doi:10.3389/fpsyt.2022.977361 PMC9468740 36111310

[28] Zhao J , Rudd A , Liu R . Challenges and potential solutions of stroke care during the coronavirus disease 2019 (COVID‐19) outbreak. Stroke. 2020;51(5):1356‐1357. doi:10.1161/STROKEAHA.120.029701 32228369 PMC7219852

[29] Man S , Xian Y , Holmes DN , et al. Association between thrombolytic door‐to‐needle time and 1‐year mortality and readmission in patients with acute ischemic stroke. Jama. 2020;323(21):2170‐2184. doi:10.1001/jama.2020.5697 32484532 PMC7267850

[30] Kamal N , Sheng S , Xian Y , et al. Delays in door‐to‐needle times and their impact on treatment time and outcomes in get with the guidelines‐stroke. Stroke. 2017;48(4):946‐954. doi:10.1161/STROKEAHA.116.015712 28228574

[31] Wang H , Camicia M , Terdiman J , Hung YY , Sandel ME . Time to inpatient rehabilitation hospital admission and functional outcomes of stroke patients. PM R. 2011;3(4):296‐304; quiz 304. doi:10.1016/j.pmrj.2010.12.018 21497314

[32] Wang H , Camicia M , DiVita M , Mix J , Niewczyk P . Early inpatient rehabilitation admission and stroke patient outcomes. Am J Phys Med Rehabil. 2015;94(2):85‐96; quiz 97‐100. doi:10.1097/PHM.0000000000000226 25569470

[33] Merkler AE , Parikh NS , Mir S , et al. Risk of ischemic stroke in patients with coronavirus disease 2019 (COVID‐19) vs patients with influenza. JAMA Neurol. 2020;77:1366‐1367. doi:10.1001/jamaneurol.2020.2730 PMC7333175 32614385

